# Conflicting Evidence between Clinical Perception and Molecular Epidemiology: The Case of Fowl Adenovirus D

**DOI:** 10.3390/ani13243851

**Published:** 2023-12-14

**Authors:** Giovanni Franzo, Giulia Faustini, Claudia Maria Tucciarone, Daniela Pasotto, Matteo Legnardi, Mattia Cecchinato

**Affiliations:** Department of Animal Medicine, Production and Health, University of Padua, Viale dell’Università, 16, 35020 Legnaro, Italy; giulia.faustini.1@phd.unipd.it (G.F.); claudiamaria.tucciarone@unipd.it (C.M.T.); daniela.pasotto@unipd.it (D.P.); matteo.legnardi@unipd.it (M.L.); mattia.cecchinato@unipd.it (M.C.)

**Keywords:** FAdV, hexon, evolution, epidemiology, phylogenesis, phylogeography

## Abstract

**Simple Summary:**

The present study demonstrates the role of intensive poultry production in the rise of fowl adenovirus (FAdV), similar to what occurred for several multifactorial viral diseases of livestock. Although effective in limiting viral circulation, the applied control strategies, likely shaped its evolution. In fact, after the development and application of FAdV vaccines, an increase in evolutionary rate was observed and several sites and regions of the Hexon protein were proven under a significant pervasive or episodic diversifying selection, especially those exposed on the viral surface and target of the host response. The notion that FAdV has increased in relevance in the last years could thus be a misleading perception related to an increased diagnostic capability and awareness of the topic. Alternatively, a limited number of flocks could be more severely affected because of the waning population immunity. Further efforts should be devoted to the acquisition of more molecular data on other geographic regions and FAdV species, also to assess the representativeness of the present results on a broader scale.

**Abstract:**

Fowl adenoviruses (FAdVs, species FAdV-A/-E) are responsible for several clinical syndromes reported with increasing frequency in poultry farms in the last decades. In the present study, a phylodynamic analysis was performed on a group of FAdV-D Hexon sequences with adequate available metadata. The obtained results demonstrated the long-term circulation of this species, at least several decades before the first identification of the disease. After a period of progressive increase, the viral population showed a high-level circulation from approximately the 1960s to the beginning of the new millennium, mirroring the expansion of intensive poultry production and animal trade. At the same time, strain migration occurred mainly from Europe to other continents, although other among-continent connections were estimated. Thereafter, the viral population declined progressively, likely due to the improved control measures, potentially including the development and application of FAdV vaccines. An increase in the viral evolutionary rate featured this phase. A role of vaccine-induced immunity in shaping viral evolution could thus be hypothesized. Accordingly, several sites of the Hexon, especially those targeted by the host response were proven under a significant pervasive or episodic diversifying selection. The present study results demonstrate the role of intensive poultry production and market globalization in the rise of FAdV. The applied control strategies, on the other hand, were effective in limiting viral circulation and shaping its evolution.

## 1. Introduction

Fowl adenoviruses are members of the genus *Aviadenovirus* and are divided into 5 species (Fowl adenovirus A-E, FAdV-A/-E), which are further classifiable in 12 serotypes [1,2]. The sequencing of the Hexon gene, the main constituent of the viral capsid, has been traditionally used to categorize aviadenoviruses in species and even at the below-species level [2,3]. In addition to the application in molecular characterization, the Hexon gene contains antigenic determinants, including neutralizing ones [4]. Different species and serotypes have been associated with variable clinical syndromes; inclusion body hepatitis (IBH) is mainly caused by strains of FAdV-2, -3, -9 and -11 serotypes (FAdV-D species) and FAdV-6, -7, -8a and -8b serotypes (FAdV-E species), while hydropericardium syndrome (HPS) is linked especially to FAdV-4 of the species FAdV-C and adenoviral gizzard erosion (AGE) to FAdV-1 (FAdV-A species), although minor exceptions are documented [1,5].

IBH-affected animals show poor growth performances, apathy, prostration and loss of conditions, with a mortality higher than 10%. Several factors are involved in disease emergence, including animal genetics, flock management, coinfecting agents and FAdV strain features [1,5]. Although its multifactorial nature complicates the quantification of FAdV impact [6], losses related to performance reduction and mortality can be considered severe [7]. FAdVs have been detected worldwide. However, in the last decades, an increased number of cases of FAdV-induced clinical outbreaks, mainly ascribable to IBH, have been reported especially in broilers up to 5 weeks of age [5,6,8,9,10,11,12,13,14,15]. Paradoxically, such an increase has been observed together with improved biosecurity in breeder flocks, which could have reduced infection risk and thus the establishment of immunity that is normally transferred maternally [5]. Nevertheless, other causes could be involved, including increased diagnostic activity and/or veterinarians’ awareness. Poultry industry intensification, movements and trades could contribute to exposing animals to different strains with variable biological and immunological features [16,17,18,19]. Viral evolution cannot be excluded either.

The intensification of the poultry sector has represented an invaluable source of high-value–low-cost animal proteins [20]. The substantial absence, with limited exceptions, of cultural and religious constraints in poultry meat and egg consumption has further prompted the success of these products all over the world [21,22]. Finally, chicken farming can significantly contribute to alternative income, poverty alleviation and food security in developing countries, especially in the small-scale village context [23,24]. Such success is frustrated by several infectious diseases that have emerged and spread over the years. While avian influenza, Newcastle disease, infectious laryngotracheitis and other major diseases drew most of the veterinarians’ and public health institutions’ attention, other infections have progressively been more and more commonly reported, causing significant economic and productive losses. Fowl adenovirus is among those detected with increasing frequency in different areas of the world [6]. Even if local epidemiological studies and trials have been performed, no study investigating the viral evolution, history and migration patterns has been currently performed.

## 2. Materials and Methods

### 2.1. Sequence Dataset Preparation

The complete collection of FAdV Hexon gene sequences was downloaded from Genbank and aligned with MAFFT version 7 [25]. Only sequences for which collection date and country were available and whose sequence quality was adequate (i.e., absence of obvious misalignment, unknown bases, premature stop codons or frameshift mutations) were maintained in the dataset. Thereafter, the region guaranteeing the best compromise between sequence length and number was selected and trimmed. A preliminary tree was reconstructed using IQ-Tree [26] selecting the substitution model with the lowest Bayesian Information Criteria (BIC) calculated using the same software. Since the purpose of the study was to investigate the history and migration patterns of FAdV circulating in the last decades, only sequences of related (i.e., within species) strains forming clusters big enough to be informative were considered for the phylodynamic analysis. Recombination occurrence was assessed using GARD [27] and the strength of the phylogenetic signal was assessed through likelihood mapping analysis implemented in IQ-Tree [26], while the temporal signal was investigated using TempEST [28].

### 2.2. Phylodynamic and Phylogeographic Analysis

The selected datasets were analyzed to reconstruct several population parameters, including time to the most recent common ancestor (tMRCA), evolutionary rate, and viral population dynamics using the Bayesian serial coalescent approach implemented in BEAST 1.10 [29]. The nucleotide substitution model was selected based on the BIC score calculated using JmodelTest2 [30]. The molecular clock was selected to calculate the marginal likelihood estimation through path-sampling and stepping-stone methods, as suggested by Baele et al. [31]. The non-parametric Bayesian Skygrid was implemented to reconstruct viral population changes over time (relative genetic diversity: effective population size∙generation time; N_e_ x τ) [32]. A discrete state phylogeographic analysis was also performed as described by Lemey et al. [33], implementing an asymmetric migration model with Bayesian stochastic search variable selection (BSSVS), allowing us to identify the most parsimonious description of the spreading process and calculate a Bayesian Factor (BF) indicative of the statistical significance of the inferred migration path between areas. Due to the sparse nature of the sequence–country combination and to obtain a more balanced dataset, countries were aggregated in macro-areas considering their spatial proximity and geopolitical factors (i.e., Africa, Asia, Europe, the Middle East, North America, Oceania and South America). Two independent runs of 200 million generations were performed. The log and tree files were merged using logcombiner after the removal of a burn in of 20%. Results were analyzed using Tracer 1.7 and accepted only if the estimated sample size (ESS) was greater than 200 and the convergence and mixing were adequate. Parameter estimation was summarized in terms of mean and 95% highest posterior density (HPD). Maximum clade credibility (MCC) trees were constructed and annotated using TreeAnnotator (BEAST 1.10 package). SpreaD3 version 0.9.6 [34] was used to calculate the BF associated with each migration route. All non-zero transition rates among countries were considered significant when the BF was greater than 10. Additional summary statistics and graphical outputs were generated using homemade R scripts [35].

### 2.3. Selective Pressure Analysis

The occurrence and pattern of selective pressures were evaluated on the Hexon gene using the non-synonymous to synonymous substitution rate calculation (dN/dS). A dN/dS higher, equal or lower than 1 suggests diversifying, neutral and purifying selection, respectively. To this purpose, all complete Hexon gene sequences from strains included in the phylodynamic analysis were downloaded when available and aligned at the codon level using the MUSCLE [36] method implemented in MEGA X [37]. Recombination breakpoint presence and location were assessed using GARD [27]. Pervasive and episodic selective pressures were analyzed using FUBAR [38] and MEME [39], implemented in HyPhy v2.3.14. [40], accounting for the partitioning due to recombination events. The significance level was set at posterior probability (PP) > 0.9 and *p*-value < 0.05 for FUBAR and MEME, respectively.

### 2.4. Homology Modeling

One strain of the clade used for the phylodynamic analysis was selected, translated at the amino acid level and used as the template for homology modeling, performed using Phyre 2 [41]. The final model was superimposed on the Hexon trimeric structure, plotted and edited using Chimera v1.16 [42].

## 3. Results

### 3.1. Dataset

A total of 573 FAdVs sequences were preliminarily selected. Based on the number, length, available metadata and distribution, only one clade, corresponding to FAdV-D strains, was big and heterogeneous enough to be considered adequate for informative phylodynamic and phylogeographic analysis. A region of 565 bp of 233 strains originating from 31 countries between 1950 and 2022 was therefore included in the final dataset (Supplementary Table S1). No evidence of statistically significant recombination events was detected by GARD in the considered region, while the phylogenetic and temporal signals were proven adequate.

### 3.2. Phylodynamic Analysis

**Figure 1:**
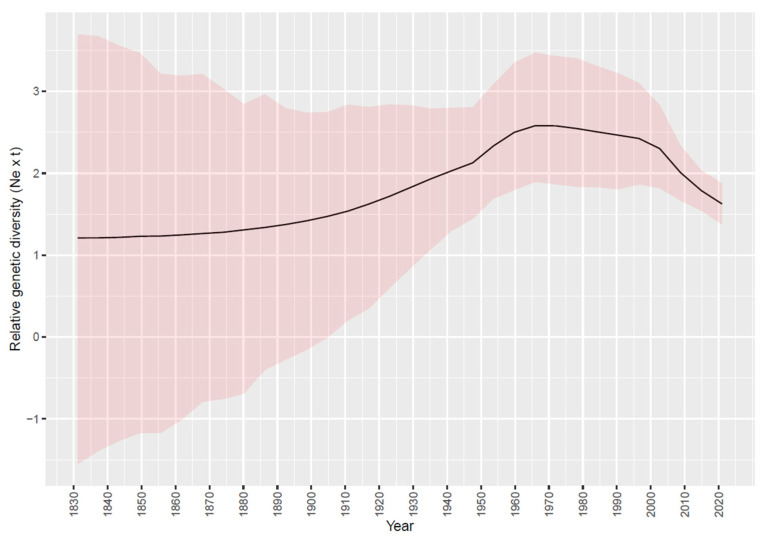
Depiction of relative genetic diversity of the selected FAdV-D clade over time in Italy. Mean values are represented as a black line, while 95HPD intervals have been displayed as red-shaded areas.

Concordant results were obtained in the two independent BEAST runs. The tMRCA of the considered clade was estimated in 1903.596 [95HPD: 1726.963–1936.241] while the evolutionary rate was 1.55 × 10^−3^ [95HPD: 4.4 × 10^−4^–5.04 × 10^−3^] substitutions/site/year. The viral population dynamics could be broadly divided into three main phases: the first one, from tMRCA to approximately the middle 1960s, was featured by a progressive, exponential increase in relative genetic diversity. This period was thereafter followed by a stabilization from the 1970s to the beginning of the new millennium when a progressive decrease was observed (Figure 1).

The phylogeographic analysis suggested a European origin, followed by progressive dispersion to other areas. Overall, a certain tendency of considered strains to cluster according to the geographic region was observed, although with several exceptions and the occurrence of multiple introduction events in the same areas as well.

**Figure 2:**
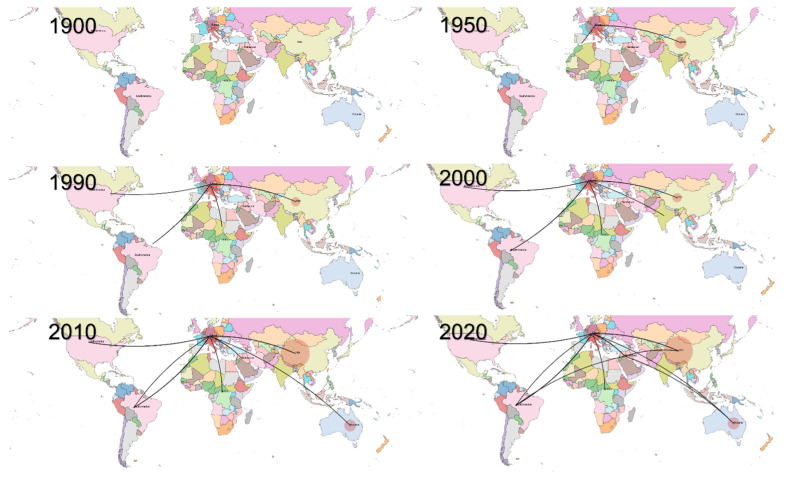
Phylogeographic reconstruction of FAdV-D clade migration over time. Each picture represents a different decade. The centroid of each macro-area has been selected as the source/destination of viral movements.

**Figure 3:**
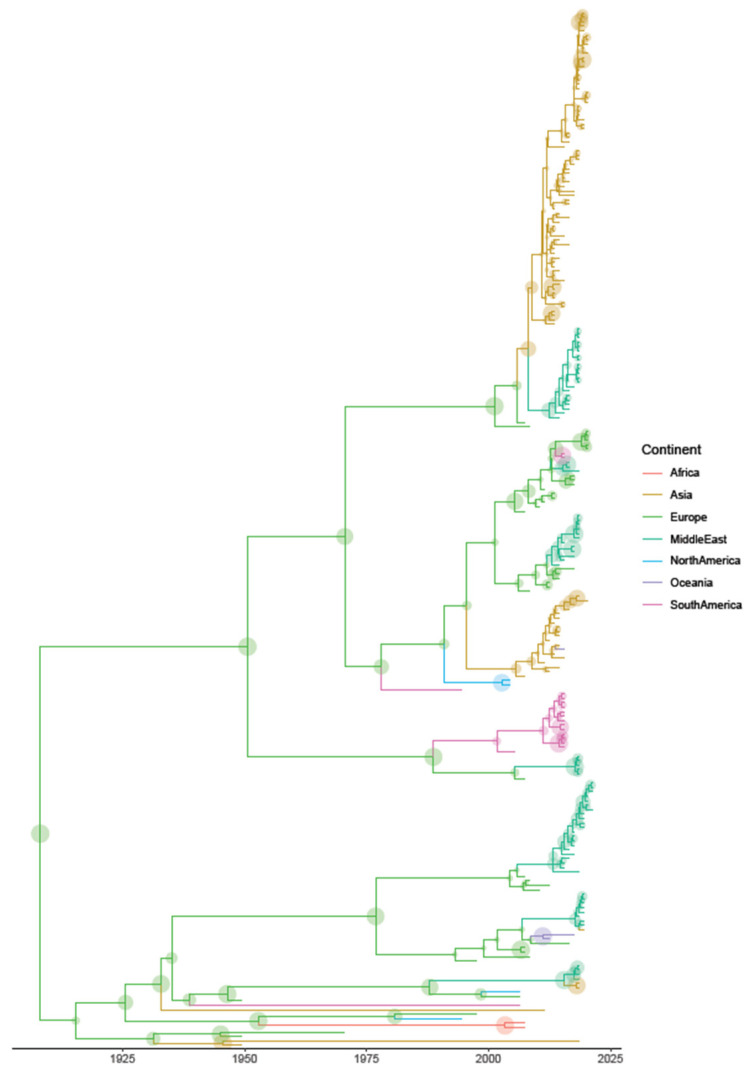
Maximum clade credibility tree of FAdV-D strains. Macro-areas where the virus ancestors were estimated to circulate have been color coded. The branch length is scaled in time (years). Circles overlapping each node are proportional to the respective posterior probability.

More in detail, after a prolonged European circulation, FAdV-D strains were progressively introduced to other areas, Asia (~1950), North and South America (~1990), Africa (~2000) and thereafter the Middle East and Oceania. Connections originating from non-European areas became clearer only in the last two decades (Figure 2 and Figure 3).

**Figure 4:**
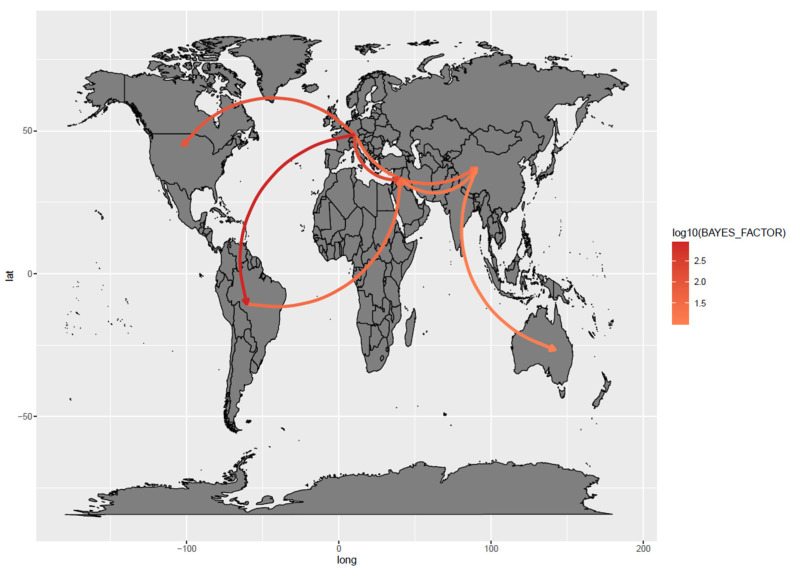
Well-supported migration paths (i.e., Bayesian factor [BF] > 10) among macro-areas, depicted as edges whose color is proportional to the log10 Bayesian factor of the inferred link.

However, a lower number of migration paths reached adequate statistical support. These involved connections from Europe to Asia, North and South America and the Middle East, which in turn was linked to Asia and South America as an exporter and importer of strains, respectively. Finally, Asia was involved in the FAdV strains’ introduction to Oceania (Figure 4).

Relationships between countries varied depending on the continent. Within Asia, clusters comprising neighboring countries like China and the Republic of Korea, and Pakistan and India were detected. Among Middle Eastern countries, most strains from Egypt, Israel, Iran, Saudi Arabia and the West Bank were interspersed, although some formed independent clusters. Several connections were finally detected within Europe, involving both neighboring and non-neighboring countries (Supplementary Figure S1). The limited number of sequences from other continents prevented the evaluation of similar patterns in other regions.

### 3.3. Selective Forces

**Figure 5:**
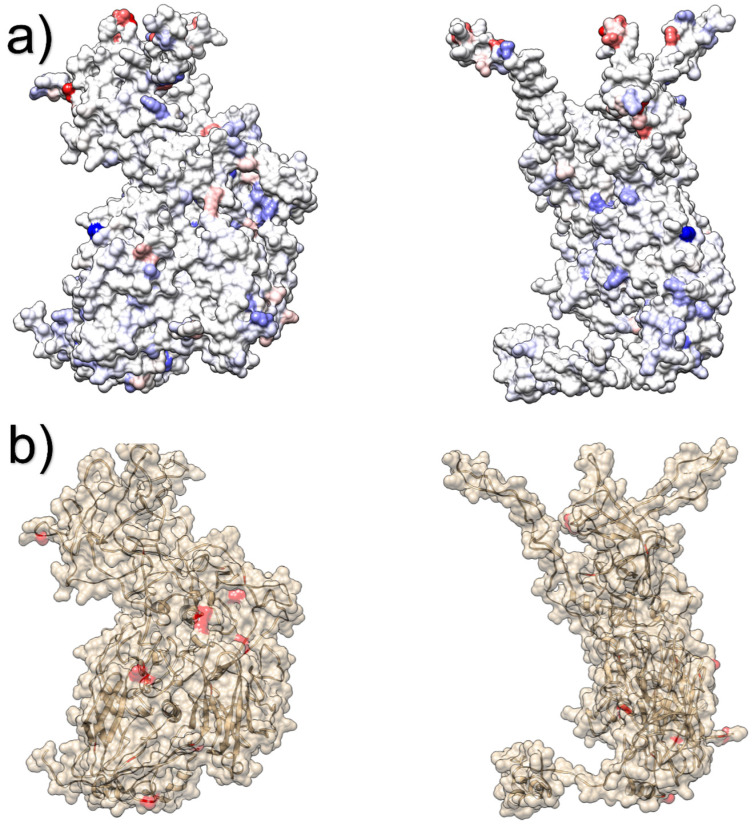
The tertiary structure of the Hexon protein is depicted from different perspectives. The surface of Figure (**a**) has been colored from red (positive values) to blue (negative values) according to the estimated dN-dS value, estimated using FUBAR. In Figure (**b**), the sites under episodic diversifying selection, estimated using MEME are reported. A more comprehensive depiction of the Hexon quaternary structure is available in Supplementary Animations S1 and S2.

Three potential recombination breakpoints were detected, dividing the alignment into 4 partitions (i.e., 1–340, 341–1527, 1528–2409 and 2410–2859). The following sites were detected under positive, diversifying selection using FUBAR: 160, 195, 199,246, 407 and 416. When the dN-dS values were plotted on the Hexon tertiary structure, most of the sites under diversifying selection were located on the protein surface, particularly on the top of the molecule surface (Figure 5a and Supplementary Animation S1). The majority of the protein was nevertheless under neutral or negative selection. A higher number of sites was reported under episodic diversifying selection—91, 188, 281, 339, 457, 549, 566, 613, 657, 671, 817, 842, 862, 922 and 949—which were more distributed in the Hexon protein (Figure 5b and Supplementary Animation S2).

**Figure 6:**
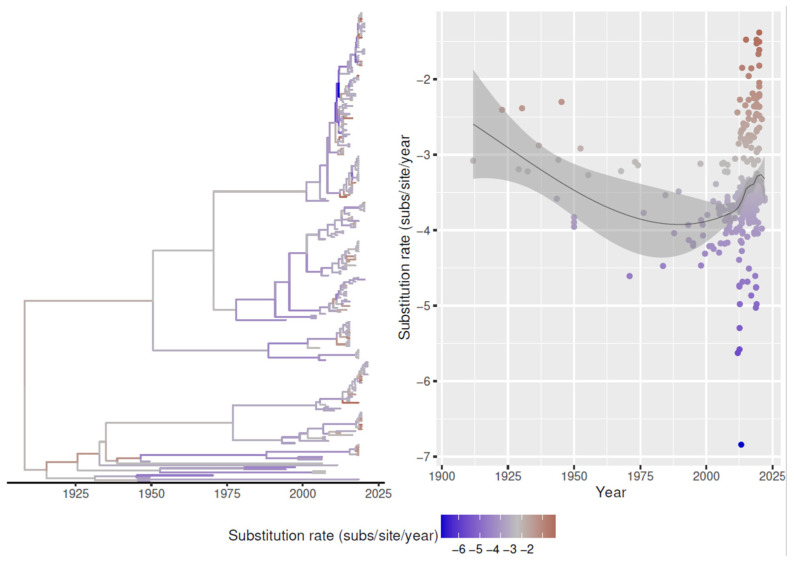
Left figure: The log10 of the evolutionary rate of each FAdV-D lineage is color-coded for each branch of the maximum clade credibility tree. Right figure: Scatter plot representing the estimated evolutionary rate (log10) of each lineage over time. A tendency line with the relative 95% confidence interval has also been superimposed.

Evolutionary rates plotted over time revealed an initial decline occurring from tMRCA to about the 1990s, followed by a progressive increase since then, although not all branches were affected by this phenomenon (Figure 6).

## 4. Discussion

The overall FAdV population dynamics and evolution have not been evaluated before on a broad, epidemiological scale. This is largely due to the poor data availability, which also represented the main limit of the present study. While different FAdV species exist, the number of available sequences suitable for phylodynamic studies is scarce for most of them. For this reason, the present investigation is focused on a subset of FAdV-D strains for which a considerable and representative number of high-quality, well-annotated sequences of the same genomic region was available.

Nevertheless, because of the biological and epidemiological similarities among FAdVs, FAdV-D was considered a suitable model for other species.

The origin of FAdV-D strains was estimated in the last few centuries (i.e., tMRCA: 1903.596 [95HPD: 1726.963–1936.241]), which is fully compatible with the first infection and disease detection [43]. However, this is in contrast with a likely co-evolution of adenovirus with their host, although occasional host jumps have been speculated [44]. The slow down of viral evolution after tMRCA might support the host jump hypothesis, requiring a rapid evolution to adapt to the new environment, followed by a progressive decrease while such adaptation is achieved. However, several other scenarios could justify the observed pattern. The progressive selection of a limited number of genetic lines for intensive poultry production could also have led to a higher homogeneity from an immune response perspective, causing lower selective pressures [45]. Similarly, the higher animal turnover could have led to a decrease in viral diversification to evade immune memory [16]. However, it must be remembered that such estimations must be carefully evaluated, especially when dealing with scarce data and ancient times for which no sequences are available.

Moreover, the evolutionary rate was 10^−3^–10^−4^ substitution/site/year, in the upper boundary of dsDNA viruses, justifying the apparent recent origin [46]. Although a recent origin followed by rapid evolution (e.g., because of host adaptation) cannot be excluded, other hypotheses must be considered. The time-dependent rate phenomenon in viruses likely plays a major role. It has been observed that viral evolutionary rate estimates are systematically negatively correlated with the timescale of rate estimation, continuously decreasing as the measurement timescale increases. In fact, rates of evolution appear to decline over time because of the combined effects of natural selection and saturation [47,48]. Therefore, although the evolutionary dynamics of viral emergence can be accurately estimated over short timeframes, their long-term evolution remains elusive [49]. Moreover, the study was performed on the sequences of the strains circulating in present times, which might represent the last descant of a broader ancient population whose majority of branches went extinguished. Therefore, the estimated tMRCA would not represent the origin of the species as a whole, but just the ancestor of the subset of strains that reaches the “sequencing era”. This hypothesis is supported by the relatively intense episodic selection acting on the Hexon gene, suggesting the presence of strong forces affecting the viral fitness and survival likelihood.

Although these intrinsic methodological and data limitations can prevent the accurate reconstruction of the ancient virus origin, a reliable depiction of recent patterns could be reached. The viral population was featured by a progressive rise and high-level circulation until about the 2000s, a pattern shared by many other livestock pathogens [50,51,52,53]. This mirrors the progressive intensification of farming that, in turn, led to an increase in animal densities, movements and trades, creating favorable conditions for viral population expansion [51,53,54]. Unfortunately, for most countries, no precise data are available regarding trades (especially over a such broad time period), preventing their evaluation and correlation with specific viral flows. Nevertheless, the overall trend and tendencies have been investigated, allowing us to at least speculate on their interaction with virus evolution and epidemiology.

Thereafter, a progressive decline occurred in the last few decades. The increase in biosecurity measures that were implemented to control other major infections likely contributed to limiting FAdV as well. Similarly, FAdV vaccines were developed starting in the late 1980s and applied in several countries or companies [5]. The study of the selective pressures highlighted a much more intense positive selection on sites exposed to the viral surface [55,56]. The role of host immunity, natural or acquired through vaccination, in driving Hexon evolution is thus likely, although none of the herein reported sites was located in previously identified epitopes [4]. Of note, several sites within the Hexon trimer were reported under episodic diversifying selection. Phases of structural adaptation of the protein in response to other forces, including compensatory mutations, can be hypothesized. The implemented vaccination protocols were extremely heterogeneous in terms of time and geographical area. Depending on the particular epidemiological scenario, observed clinical syndromes and vaccine availability, vaccines based on different species and/or serotypes were applied, whose cross-protection is typically incomplete or unknown [57]. Because of all these uncertainties, the precise contribution of vaccination in shaping FAdV epidemiology and evolution could not be reliably investigated. However, the progressive increase in the estimated evolutionary rate observed since approximately the 1990s, when vaccines started to be developed and applied [5], could suggest at least a contribution of this force in affecting FAdV evolution.

Interestingly, reports of FAdV outbreaks have become more frequent in recent years and it has been tentatively and paradoxically attributed to improved biosecurity measures in breeders’ farms, leading to a lower maternal immunity transfer [5]. The study of the underlying population dynamics conflicts with the clinical perception since the last decades featured a progressive decrease in FAdV-D circulations. Different, non-conflicting hypotheses can be advocated. At first, an increase in awareness and diagnostic activity, combined with more effective diagnostic tools, could have led to an apparent prevalence increase. Also, in this case, no precise data of the diagnostic activity intensity in different countries could be obtained. Nevertheless, with the increasing development, availability and decreased costs, it is well established that diagnostic opportunities expanded remarkably in veterinary medicine all over the world as well, although still with a high heterogeneity depending on the considered area. At the same time, lower viral circulation, although beneficial for the overall population, could have exposed a minority of flocks, especially those with suboptimal management, to an increased risk of developing more severe outbreaks whose typical clinical picture led to an easier diagnosis. Based on this evidence, the implemented control strategies were proven effective and further efforts should be directed toward their enforcement in realities still suffering from clinical outbreaks.

The phylogeographic analysis revealed a continent and (to a lesser extent) country-based geographical clustering, which testifies to the rare occurrence of long-distance dispersal events. However, in parallel with the intensification of farming and animal trade, new strain introduction became more common and effective in the infection establishment in new areas. Thereafter, local dispersal was more efficient, at least in certain regions where close geographical, economic or socio-political relationships are in place, like those observed among neighboring Asian or Middle Eastern countries. Among European countries, viral dispersal was even more common, likely because of the European Single Market facilitating strain exchange as reported for other poultry pathogens [53,58,59]. On the contrary, countries like Iran that have been commercially isolated from most trade exchanges for geopolitical reasons appeared protected from recent introduction events and the detected strains were likely the result of past introduction events followed by local evolution [60]. Therefore, intense among-countries contacts are probably necessary to allow an effective strain introduction and infection establishment. Whether this can be extrapolated to other geographic regions remains unknown. Unfortunately, the limited sequence obtained for most continents and the close genetic similarity prevent the precise reconstruction of additional migration paths.

## 5. Conclusions

The present study allowed the reconstruction of the history and epidemiology of a major FAdV-D clade, supporting the effect of poultry farm development in enhancing the success of such infection and, on the other hand, the efficacy of adequate control measures in constraining its circulation. The notion that FAdV has increased in relevance in the last years could be a misleading perception related to an increased diagnostic capability and awareness of the topic. Alternatively, a limited number of flocks could be more severely affected because of the waning population immunity. Nevertheless, when considered globally, the benefits of applied control strategies seem to largely outclass the supposed shortcomings. Because of the biological and epidemiological similarities among FAdVs, we consider FAdV-D a potential model for other species. Nevertheless, we must stress that any inference of the present results beyond the experimentally investigated boundaries must be taken with caution and dedicated sequencing activity and epidemiological studies should be performed to assess among-species differences in epidemiological patterns. The lack of high-resolution data on poultry farming management, control strategies, trades and diagnostic activity prevents the specific evaluation of the effect size of the different factors investigated in the present study, allowing only a qualitative assessment. In the “Big Data” era further efforts should thus be paid to acquire more epidemiological and molecular data and extend them to other geographic regions and FAdV species, to refine the results and assess their representativeness on a broader scale.

## Data Availability

Data are contained within the article and supplementary materials.

## Supplementary Materials

The following supporting information can be downloaded at: https://www.mdpi.com/article/10.3390/ani13243851/s1, Figure S1: Time scaled, maximum clade credibility (MCC) trees FAdV-D strains. The tree branches have been color-coded according to the macro-areas location predicted with the highest posterior probability. Animation S1: Quaternary structure of the Hexon trimer, obtained through homology modeling. The different chains have been colored to display different information. In grey, the overall tertiary structure of the Hexon protein is depicted. The amino acid position is depicted from yellow (N-terminal) to red (C-terminal) in the second chain. Finally, the surface of the last chain has been color coded from red (positive values) to blue (negative values) according to the estimated dN-dS value, estimated using FUBAR. Animation S2: Tertiary structure of the Hexon protein. The sites under episodic diversifying selection, estimated using MEME, are reported in red on the protein surface and underlying ribbon structure. Table S1: List (accession numbers) of the sequences used in the present study and relative metadata (geographical location and collection year).
