# Supplementary figures and images for: Conflicting Evidence between Clinical Perception and Molecular Epidemiology: The Case of Fowl Adenovirus D
Source: Animals (Basel). 2023 Dec 14;13(24):3851. doi: 10.3390/ani13243851 (PMC10741239; doi:10.3390/ani13243851)

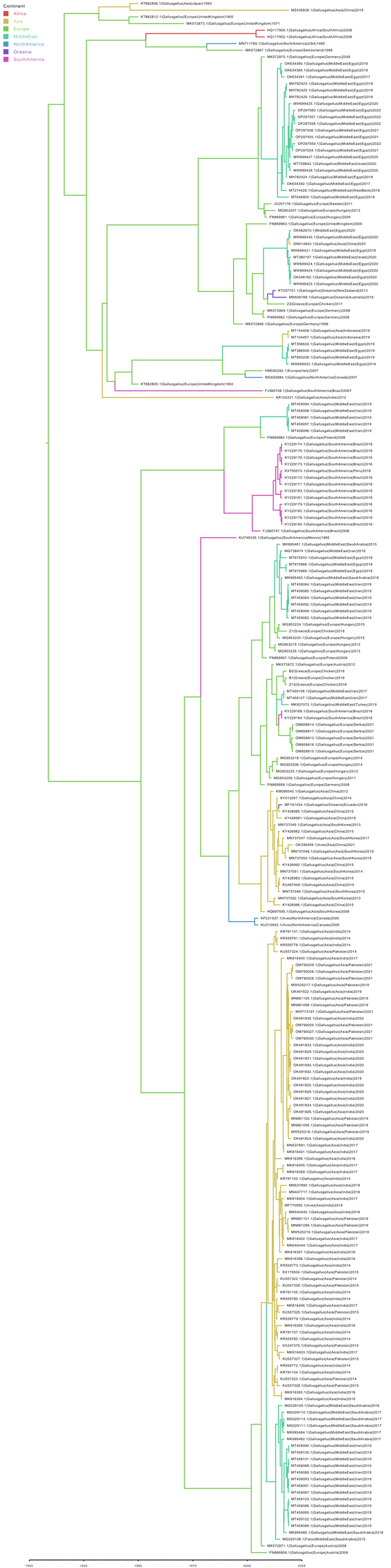

Supplement: Supplementary file 1 [file animals-13-03851-s001.zip › Supplementary figure S1.pdf]
